# The impact of illness perception on quality of life in lung cancer chemotherapy patients: mediating effect of fear of progression

**DOI:** 10.3389/fpsyt.2025.1704198

**Published:** 2025-11-24

**Authors:** Fan Xu, Shaoju Xie, Qiao Li, Xiaoli Zhong, Jiquan Zhang

**Affiliations:** 1 Oncology Department, Deyang People’s Hospital, Deyang, Sichuan, China; 2 Nursing Department, Deyang People’s Hospital, Deyang, Sichuan, China; 3 Nephrology Department, Deyang People’s Hospital, Deyang, Sichuan, China

**Keywords:** lung cancer, illness perception, quality of life, fear of progression, mediation effect

## Abstract

**Background:**

Quality of life (QoL) in lung cancer chemotherapy patients has been a key concern for researchers, and there are no studies examining the relationship between illness perception(IP), fear of progression(FoP), and QoL in lung cancer chemotherapy patients. This remains an understudied topic.

**Objective:**

Exploring the mediating effect of FoP between IP and QoL in lung cancer chemotherapy patients.

**Methods:**

From January to June 2024, 390 lung cancer chemotherapy patients were recruited through convenience sampling from the outpatient clinics and inpatient wards of the Department of Oncology at a tertiary Grade-A hospital in Deyang, China. Participants completed a battery of instruments comprising a general information questionnaire, the Brief Illness Perception Questionnaire (BIPQ), the Fear of Progression Questionnaire–Short Form (FoP-Q-SF), and the Functional Assessment of Cancer Therapy–Lung (FACT-L). Data were analyzed with SPSS 26.0 to examine associations among IP, FoP, and QoL. The mediating role of FoP in the relationship between IP and QoL was tested with the PROCESS 4.1 macro.

**Results:**

Total IP score (44.71 ± 9.47), FoP score (39.35 ± 6.79), and total QoL score (66.43 ± 23.67) in lung cancer chemotherapy patients; Pearson’s correlation analysis showed that IP was negatively correlated with QoL (r=-0.401, P<0.001) and positively correlated with FoP (r=0.363, P<0.001); FoP was negatively correlated with QoL (r=-0.319, P<0.001); Mediation analysis revealed that FoP partially mediated the relationship between IP and QoL, accounting for 18.5% of the total effect (indirect effect = -0.065, 95% CI [-0.107, -0.027]).

**Conclusion:**

Lung cancer chemotherapy patients’ IP can directly affect the QoL, and can also indirectly affect the QoL through FoP. Therefore, in clinical practice, we should focus on the IP and FoP of lung cancer chemotherapy patients, and provide effective psychological guidance and clinical intervention when necessary, while medical institutions can take some targeted measures to improve the negative emotions and psychological cognition of patients and improve the QoL.

## 1 Introduction

Global cancer statistics for 2022 indicate that there were 2.48 million new cases of lung cancer, accounting for 12.4% of all new cancer cases, making it the most commonly diagnosed cancer worldwide (1). The insidious onset of lung cancer, coupled with the lack of effective screening methods and non-specific early symptoms, often results in patients presenting at advanced stages of the disease (2).Chemotherapy, a cornerstone of lung cancer treatment, can prolong patient survival and control cancer recurrence and metastasis. However, long-term chemotherapy may lead to a series of adverse reactions, such as pain, fatigue, nausea, alopecia, diarrhea, and myelosuppression, which can cause patients to worry about their prognosis, lose confidence in treatment, and impact their quality of life(QoL) (3, 4). The world health organization (WHO) defines QoL as an individual’s perception of their position in life within the context of the culture and value systems in which they live, relative to their goals, expectations, standards, and concerns. This definition reflects a person’s experience of their physical condition, psychological functioning, social capabilities, and overall well-being (5, 6). Research has shown that QoL is a significant health outcome and the ultimate goal of all health interventions (7). Therefore, quantifying QoL and analyzing its potential influencing factors are crucial for optimizing health strategies and enhancing individuals’ physical and mental health levels.

Illness perception (IP) refers to an individual’s understanding of their disease, defined as the process by which patients interpret and analyze their current symptoms or disease through prior knowledge and experience (8, 9). Research shows that IP acts as a central mechanism in patients’ self-management by impacting coping strategies, which in turn directly influence disease attitudes and lifestyle decisions (10, 11).IP has been shown to have a significant relationship with QoL across various disease groups. A study of chemotherapy patients with hematologic malignancies revealed that IP negatively predicts QoL (12). Similarly, in stroke patients, IP was found to be significantly and negatively associated with QoL (13). Additionally, research has found that IP, fear-related emotions, and negative physical conditions (e.g., fatigue, pain) are significant determinants of QoL in cancer patients (14, 15).

Fear of progression (FoP) is a conscious, reactive fear stemming from an individual’s confrontation with disease and its biological, psychological, and social repercussions, including the dread of disease recurrence (16). Research indicates that in bladder cancer patients, IP significantly influences FoP, with a strong positive correlation between the perceived severity of symptoms and FoP levels (17). Similarly, Corter et al.’s study involving 153 breast cancer patients revealed a significant positive link between IP and FoP (18).In lung cancer patients, FoP prevalence ranges from 37% to 77.93% (19–21), and this fear can persist for years post-diagnosis (22).Persistent fear of disease is closely associated with heightened anxiety and depression (23). If not properly addressed and managed in a timely manner, long-term excessive FoP may lead to maladaptive behaviors such as healthcare avoidance, potentially accelerating disease progression (24). Existing studies have shown that FoP can have a significant impact on patients’ QoL (25, 26). Othman’s research further suggests that FoP can influence QoL by affecting disease management strategies (27).

The common-sense model of self-regulation(CSM), introduced by Leventhal et al. in 1980 (28, 29), offers a comprehensive framework for understanding how patients use their lay beliefs to initiate and sustain self-regulatory processes, gaining deeper insights into and effectively managing their illnesses. It provides a robust theoretical basis for analyzing the interplay between illness representations, coping behaviors, and health outcomes. The model posits that when patients face health threats, they form IPs at cognitive and emotional levels, which influence emotional responses and coping behaviors, dynamically affecting prognosis, QoL, and social functioning. Research has explored the mediating role of FoP between IP and QoL in other diseases. For instance, a study on patients with interstitial lung disease found that FoP mediated the relationship between IP and QoL, with an indirect effect of 0.121 and a mediation effect accounting for 26.36% of the total effect (30).

Therefore, based on previous research findings, we proposed the following hypotheses.

Hypothesis 1: IP had a direct path on QoL.
Hypothesis 2: FoP mediated the relationship between IP and QoL.

We conducted this study due to limitations in previous research on QoL in lung cancer chemotherapy patients. First, prior studies primarily focused on health-related QoL (31–34), without comprehensive consideration of overall QoL. Second, while the relationships among IP, FoP, and QoL have been explored in groups such as cervical cancer and interstitial lung disease patients (30, 35), it is important to note that compared to cervical cancer patients who focus on fertility loss, sexual dysfunction, and the reconstruction of female identity (36), and interstitial lung disease patients who adapt to disease uncertainty and the loss of daily capabilities (30), lung cancer chemotherapy patients may have unique psychological mechanisms due to treatment side effects (e.g. intractable nausea and vomiting, severe fatigue) and prognostic uncertainty.

**Figure 1:**
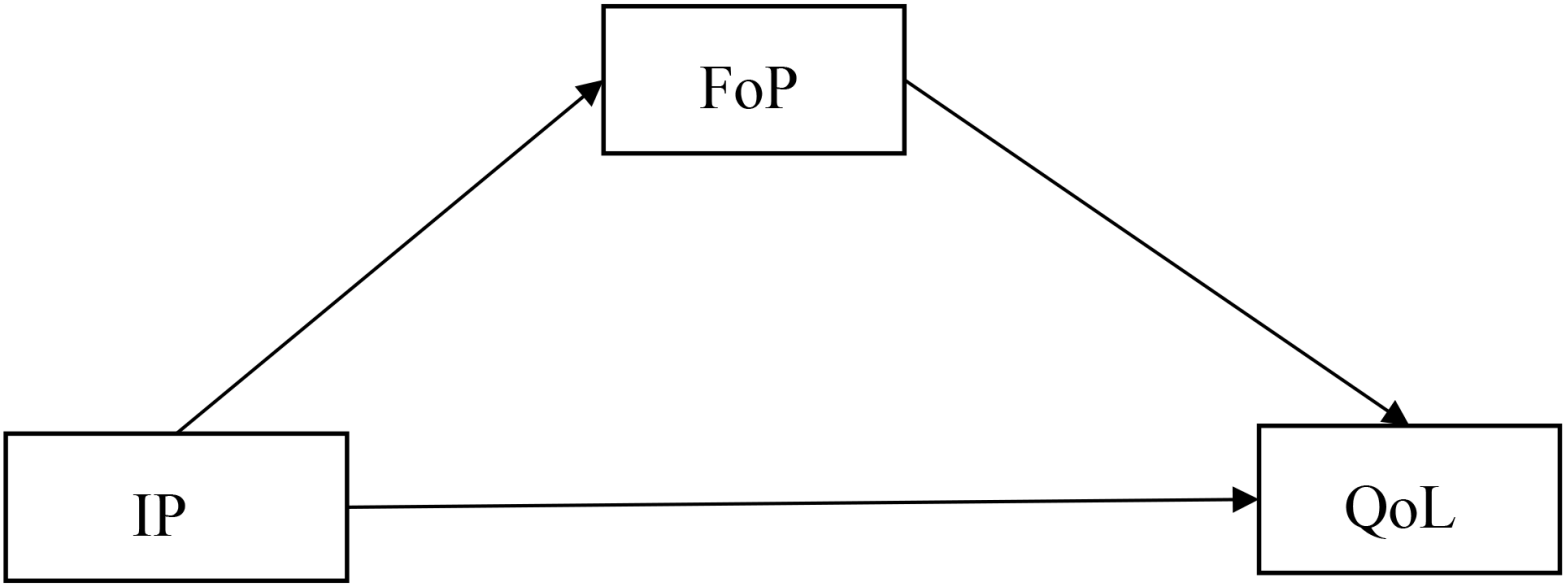
Hypothesized theoretical model. IP, illness perception; QoL, quality of life; FoP, fear of progression.

In light of the above, this study, grounded in the CSM framework, developed the following hypotheses (see **Figure 1** for the hypothesized model). The study aimed to: (1) Examine the relationship between IP and QoL in lung cancer chemotherapy patients; (2) Confirm the mediating role of FoP between illness perception and QoL in this patient population.

## 2 Materials and methods

### 2.1 Study design and participants

A cross-sectional design was employed. From January to June 2024, we recruited participants at a tertiary hospital in Deyang City. Lung cancer chemotherapy patients were given the general information questionnaire, the Brief Illness Perception Questionnaire (BIPQ), the Fear of Progression Questionnaire-Short Form (FoP-Q-SF), and the Functional Assessment of Cancer Therapy-Lung (FACT-L) scale to complete, which took 20–30 minutes.

### 2.2 Participants

Inclusion criteria: (1) patients diagnosed with primary bronchopulmonary cancer and admitted for chemotherapy after histopathologic confirmation; (2) consciousness and no communication barriers; (3) age ≥18 years; (4) expected survival time ≥6 months; and (5) voluntary participation. Exclusion criteria: (1) scheduled surgery post-diagnosis; (2) mental or cognitive disorders; (3) lack of awareness of their cancer diagnosis; and (4) comorbidities involving serious heart, liver, kidney, or other organ diseases.

The sample size was calculated using the formula n=[u_α_*σ/δ]² for cross-sectional surveys, with α=0.05, u^α^ =1.96, tolerance error δ = 2.0 (37), and standard deviation σ= 15.554 (38). This yielded a minimum of 232 participants; accounting for a 20% attrition rate, the target sample was 255. The study recruited a total of 400 patients, with 390 ultimately included in the analysis. Ten participants were excluded due to refusal to participate.

### 2.3 Ethics statement

Ethical approval for this study was granted by Deyang People’s Hospital (Code:202204058K01). Prior to participation, a trained investigator informed patients about the study’s purpose, importance, and procedural instructions. Anonymity was assured to protect privacy, and informed consent was obtained from all participants.

### 2.4 Measures

#### 2.4.1 Demographic characteristics and disease-related information

A general information questionnaire collected demographic and disease-related data, including age, gender, number of children, marital status, education, religion, occupation, monthly household income, residence, cancer stage, and comorbidities. Indicator categorization is shown in **Table 1**.

**Table 1:** Demographic characteristics and disease-related information of lung cancer chemotherapy patients.

Variable		N(%)	QoL(Mean ± SD)	*t/F*	*P*	LSD
Gender	Male	282(72.31)	65.72 ± 24.16	-0.966^*^	0.334	
	Female	108(27.69)	68.31 ± 22.34			
Age(Year)	18~44^a^	23(5.90)	77.13 ± 23.34	3.992	**0.019**	a>c
	45~59^b^	183(46.92)	67.86 ± 23.15			
	≥60^c^	184(47.18)	63.67 ± 23.85			
Number of children	None	4(1.03)	68.50 ± 25.44	0.412	0.744	
	1	255(65.38)	66.98 ± 23.02			
	2	95(24.36)	64.18 ± 24.41			
	≥3	36(9.23)	68.25 ± 26.61			
Marital status	Married	326(83.59)	66.44 ± 23.22	0.026^*^	0.989	
	Single	28(7.18)	66.32 ± 29.40			
Education level	Primary and below	152(38.97)	65.26 ± 26.33	1.539	0.216	
	Middle school	199(51.03)	66.12 ± 22.06			
	College and above	39(10)	72.62 ± 20.05			
Religion	Yes	23(5.9)	66.00 ± 19.95	-0.090^*^	0.928	
	No	367(94.1)	66.46 ± 23.91			
Occupational status	Employed	57(14.62)	63.42 ± 24.80	1.762	0.173	
	Retired	169(43.33)	68.94 ± 22.55			
	Unemployed	164(42.05)	64.90 ± 24.29			
Per capita monthly household income(Yuan)	<3000^a^	232(59.49)	64.38 ± 23.23	5.338	**0.001**	a,b,c<d
	3000-4999^b^	85(21.79)	65.02 ± 24.06			
	5000-9999^c^	54(13.85)	71.02 ± 20.21			
	≥10000^d^	19(4.87)	84.79 ± 28.33			
Place of residence	City	221(56.67)	67.78 ± 23.62	1.063	0.347	
	Townships	48(12.31)	66.65 ± 24.85			
	Villages	121(31.03)	63.88 ± 23.29			
Clinical stage of cancer	I^a^	25(6.41)	76.24 ± 29.32	4.975	**0.002**	a,b,c>d a>c
	II^b^	85(21.79)	71.20 ± 26.40			
	III^c^	177(45.38)	66.28 ± 21.03			
	IV^d^	103(26.41)	60.38 ± 22.77			
Comorbidity with other diseases	Yes	218(55.90)	64.85 ± 21.64	1.459^*^	0.145	
	No	172(44.10)	68.44 ± 25.95			

*Indicates independent samples t-test. IP, illness perception; QoL, quality of life; FoP, fear of progression, LSD, least significant difference. a, b, c and d represent different category groups. Bold values indicate P< 0.05.

#### 2.4.2 Measurement of QoL

The Functional Assessment of Cancer Therapy-Lung (FACT-L) scale, developed by Cella et al. (39)and adapted to Chinese by Wan et al. (40), assesses QoL in Chinese lung cancer patients (41). The scale includes 36 items across five dimensions: Physical Well-Being (PWB), Social/Family Well-Being (SWB), Emotional Well-Being (EWB), Functional Well-Being (FWB), and the Lung Cancer Subscale (LCS). Each item is scored on a 5-point Likert scale (0–4), for a total score of 0-144, with higher scores indicating better QoL. The scale’s Cronbach’s alpha was 0.758, and in this study, it was 0.926.

#### 2.4.3 Measurement of IP

The Brief Illness Perception Questionnaire (BIPQ), developed by Broadbent et al. (9), assesses patients’ perceptions of their illness through three dimensions: cognition, emotion, and understanding, via nine items. Items 1 to 8 are scored on a scale of 0 to 10, with items 3, 4, and 7 reverse-scored. The total score is the sum of all items, where a higher score indicates a greater perceived threat of the disease. Item 9 is an open - ended question asking participants to identify the three main causes of their disease, this study did not incorporate quantitative analysis. The scale showed good reliability with a Cronbach’s α of 0.84 in a study of lung cancer patients (42) and 0.823 in this research.

#### 2.4.4 Measurement of FoP

The Fear of Progression Questionnaire-Short Form (FoP-Q-SF) was developed by German researcher Mehnert and colleagues in 2006 based on the Fear of Progression Questionnaire (43), and it includes 12 items with a Cronbach’s α of 0.87. Wu et al. (44) adapted and revised it for China. The scale consists of two dimensions: the Physical Health Concerns and the Social/Family Concerns. It employs a Likert 5-point scale, r scored from 1 to 5 in ascending order: never, rarely, sometimes, often, always, with total scores ranging from 12 to 60. Higher scores represent a higher fear of disease progression. A total score of 34 or above indicates clinically significant psychological dysfunction requiring intervention. The cross-cultural applicability of the FoP-Q-SF has been validated in studies across different countries, with a Cronbach’s α coefficient of 0.83 in a study conducted in Turkey (45). In this study, the scale demonstrated a Cronbach’s α of 0.854.

### 2.5 Data collection and quality control

To guarantee uniform data collection, all participants engaged in standardized training, which encompassed questionnaire content, patient communication tactics, and the prevention of leading questions. Before the survey began, researchers clarified the study’s purpose and details to participants via a consistent script. Once informed consent was secured from patients and their families, the questionnaires were distributed on-site. Throughout the survey, one researcher promptly clarified patients’ doubts, while another monitored the proceedings. Upon completion, questionnaires were immediately collected and scrutinized for any omissions or errors to boost data quality. Any missing objective data were supplemented by referring to medical records. If, after this supplementation, the missing data rate still exceeded 10%, the questionnaire was discarded. For questionnaires with a missing data rate of 10% or below, missing quantitative variables were replaced with the mean value, and missing categorical variables were filled in with the mode.

### 2.6 Data analysis

Data were analyzed using SPSS 26.0, with a significance level set at α = 0.05. Normality was assessed via P-P plots and histograms. Quantitative data are presented as mean ± SD, and categorical data as frequency and percentage. Differences in QoL across groups were tested using t-tests and one-way ANOVA, with (least significant difference)LSD *post-hoc* tests for pairwise comparisons. Pearson correlation analysis was used to evaluate the relationships between IP, fear of disease progression, and QoL. Harman’s single-factor test via exploratory factor analysis was employed to assess common method bias. After standardizing the data, Hayes’ PROCESS 4.1 Model 4 was used for mediation analysis, with the significance of the mediation effect tested via bias-corrected percentile Bootstrap method. A 95% CI not containing zero indicated a statistically significant effect (46).

## 3 Results

### 3.1 Demographic characteristics and disease-related information

A total of 390 patients were included in the final analysis. Group differences in patient characteristics and QoL are presented in **Table 1**. The mean age of patients was 59.11 years (SD = 11.37; range 28–83); Male patients constituted 72.31% of the study cohort, 47.18% were aged ≥60 years, 65.38% had one child, 83.59% were married, “51.03% had Middle school education the vast majority (94.1%) had no religious affiliation, 43.33% were retired, 59.49% of patients had a per capita monthly household income below ¥3,000, 56.67% resided in city, nearly half (46.92%) had stage III cancer, and over half (55.9%) had comorbidities. Age[F(2, 387) = 3.992, p = 0.019, η²= 0.020], per capita monthly household income[F(2, 387) = 3.992, p = 0.019, η²= 0.020], and cancer stage[F(2, 387) = 3.992, p = 0.019, η²= 0.020] were significant predictors of QoL in lung cancer chemotherapy patients.

### 3.2 Correlation analysis of IP, FoP and QoL in lung cancer chemotherapy patients

Correlations, means, and standard deviations of the related variables are presented in **Table 2**. Correlation analysis revealed that the total IP score in lung cancer chemotherapy patients was positively correlated with the total FoP score (r = 0.363, *P* < 0.01) and negatively correlated with the total QoL score (r = -0.401, *P* < 0.01). Additionally, the FoP score was negatively correlated with the total QoL score (r =-0.319, *P* < 0.01), as shown in **Table 2**.

**Table 2:** Correlation analysis of IP, FoP and QoL in lung cancer chemotherapy patients.

Variable	Mean	SD	IP	FoP	QoL
IP	44.71	9.47	1		
FoP	39.35	6.79	0.363^**^	1	
QoL	66.43	23.67	-0.401^**^	-0.319^**^	1

^**^P < 0.01. IP, illness perception; QoL, quality of life; FoP, fear of progression.

### 3.3 Analysis of the mediating effect of FoP between IP and QoL in lung cancer chemotherapy patients

Harman’s single-factor analysis was used to test for common method bias. 12 factors with eigenvalues >1 were found. The first factor explained 21.56% of variance, below the 40% threshold, indicating no severe common method bias (47). Furthermore, with QoL as the dependent variable, a multiple regression analysis was conducted incorporating statistically significant variables identified in the univariate and correlation analyses. The results of the multicollinearity diagnostics showed that the tolerance values for all variables ranged from 0.815 to 0.972, while the variance inflation factors (VIF) were between 1.029 and 1.227, all significantly below the threshold of 10. This indicates that there were no evident multicollinearity issues among the independent variables.

All variables were standardized and mediation analysis was performed using Hayes’ PROCESS 4.1 Model 4. Results showed that after controlling for age, per capita monthly household income, and cancer stage, IP in lung cancer patients negatively predicted QoL (β = -0.351, t = -7.392, *P* < 0.001) and positively predicted FoP (β = 0.348, t = 7.076, *P* < 0.001). Additionally, FoP negatively predicted QoL (β = -0.186, t = -5.773, *P* < 0.001). These findings are detailed in **Table 3**.

**Table 3:** Testing the mediating effect of FoP between IP and QoL.

Variable		Model 1		Model 2		Model 3	
		QoL		FoP		QoL	
		β	t	β	t	β	t
Independent variable	IP	-0.351	-7.392***	0.348	7.076***	-0.286	-5.773***
Mediating variable	FoP	–	–	–	–	-0.186	-3.844***
Control variable	Age	-0.178	-2.312*	0.148	1.859	-0.150	-1.980*
	Per capita monthly household income	0.117	2.239*	-0.036	-0.664	0.111	2.147*
	Clinical stage of cancer	-0.139	-2.563*	0. 012	0.215	-0.137	-2.567*
	*R*	0.443		0.375		0.476	
	*R^2^*	0.197		0.140		0.226	
	*F*	23.556		15.711		22.475	

^*^P<0.05; ^***^P<0.001. IP, illness perception; QoL, quality of life; FoP, fear of progression.

**Figure 2:**
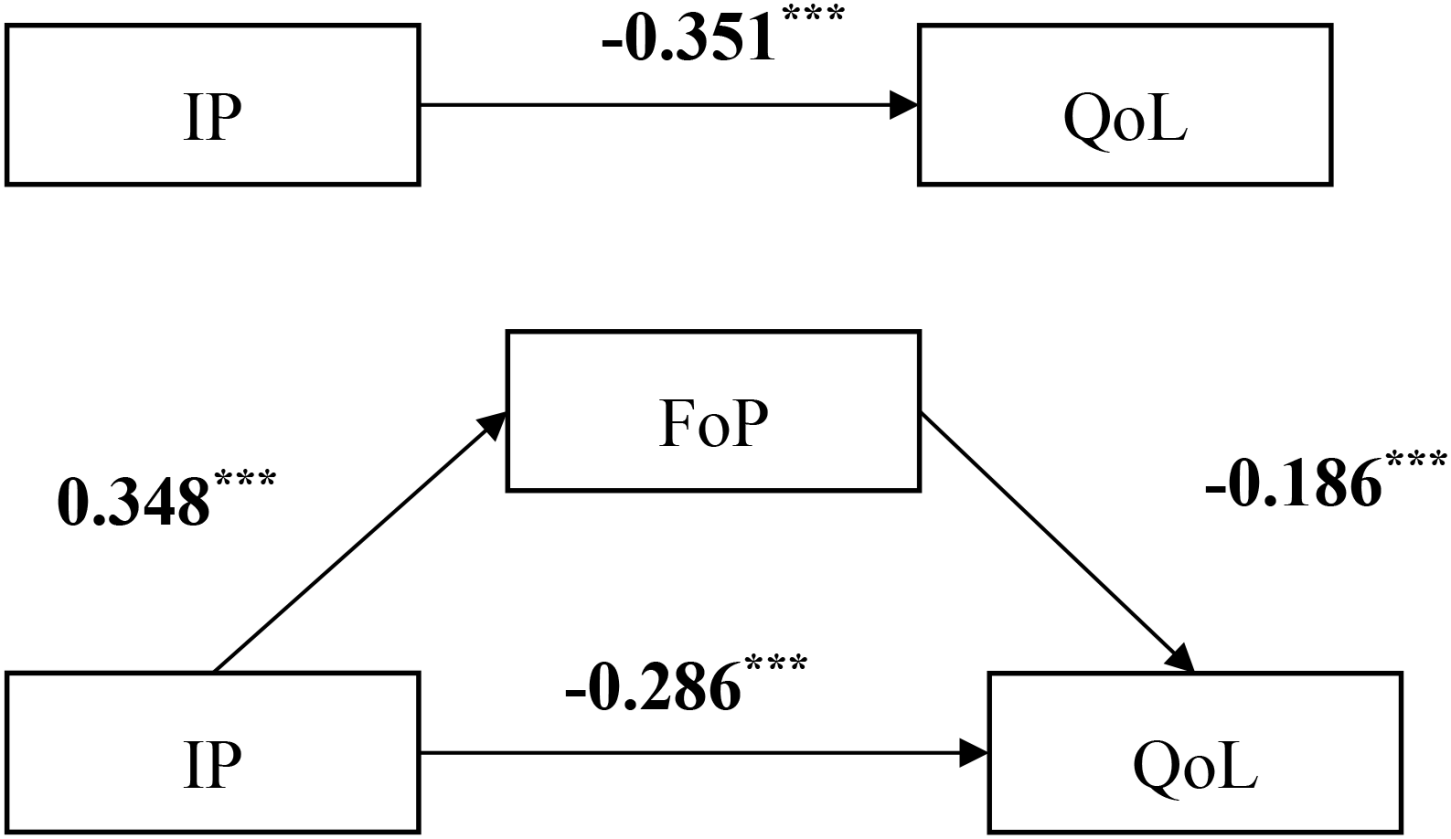
Modeling the mediating effect of IP on QoL through FoP. ^***^Indicating the coefficient of the path is significant. IP, illness perception; QoL, quality of life; FoP, fear of progression.

Bootstrap analysis with 5000 resamples was conducted to assess the mediating effect (48), with a 95% confidence interval(CI). The results indicated that the standardized total effect of IP on QoL was -0.351 (95% CI [-0.445, -0.258]). The standardized direct effect was -0.286 (95% CI [-0.384, -0.189]), excluding zero and accounting for 81.5% of the total effect. The standardized indirect effect mediated by fear of disease progression was -0.065 (95% CI [-0.106, -0.027]), also excluding zero and constituting 18.5% of the total effect. These findings suggest that FoP plays a partial mediating role in the relationship between disease perception and QoL, as illustrated in **Figure 2** and **Table 4**.

**Table 4:** Bootstrap based mediation effect test.

Variable	Effect	BootSE	95%CI	Ratio of effect
Total path of IP on QoL	-0.351	0.048	-0.445 ~ -0.258	–
Direct path of IP on QoL	-0.286	0.050	-0.384~ -0.189	81.5%
Indirect path of IP on QoL	-0.065	0.020	-0.107 ~ -0.027	18.5%

IP, illness perception; QoL, quality of life; FoP, fear of progression.

## 4 Discussion

To our knowledge, this is the first study to investigate the mediating role of FoP in IP and QoL among lung cancer chemotherapy patients. As demonstrated herein, our findings are supported by the self-regulation common sense model and validate our prior hypothesis that IP positively correlates with QoL in lung cancer chemotherapy patients, with FoP acting as a mediator. In simple terms, patients perceiving a greater symptom burden tend to experience heightened FoP, which often leads to poorer QoL. This finding offers a novel perspective for improving patient QoL, suggesting that alleviating symptom burden may mitigate FoP, thereby helping patients engage more positively with their illness and potentially improve their prognosis.

In this study, lung cancer chemotherapy patients had a QoL score of 66.43 ± 23.67, lower than the 79.90 ± 15.84 reported in a Chinese study on lung cancer patients receiving immunotherapy (49). These differences may stem from variations in patient characteristics such as age, socioeconomic status, disease severity, and comorbidities. In our research, age, per capita monthly family income, and cancer clinical stage emerged as key predictors of QoL. Age exerts a detrimental influence on QoL, consistent with prior research findings (50). It is plausible that as patients age, their health status and capacity for self-care diminish, consequently leading to a decline in their QoL. Moreover, per capita monthly household income exerts a positive influence on QoL. Research indicates that economic circumstances serve as a protective factor for cancer patients’ QoL (51, 52). Patients with higher incomes can afford superior medical services, leading to better prognosis and enhanced QoL. Concurrently, cancer clinical staging exerts a negative impact on QoL, consistent with prior studies (53). Patients with earlier clinical stages experience milder symptoms and exhibit better physical and psychological conditions; conversely, those with later stages endure more severe symptoms, which significantly impair their QoL (54). Research indicates that elderly, low-income, and late-stage patients should be identified as high-risk groups for impaired QoL and incorporated into priority screening and monitoring systems. Secondly, given that economic factors constitute a significant protective variable for QoL, it is recommended that clinicians routinely assess patients’ financial toxicity during initial consultations and follow-ups, providing targeted support such as simplified treatment regimens and financial assistance for those experiencing economic hardship. Finally, advanced-stage patients should receive early integrated symptom management and palliative care. Multidisciplinary collaboration can alleviate their physical and psychological symptom burden, thereby delaying the decline in QoL.

Disease perception and QoL were significantly negatively correlated, which was consistent with hypothesis 1, namely, the more negative the patients’ disease perception was, the worse their QoL would be, and this was in line with the findings of similar studies (55, 56).Disease perception reflects patients’ subjective understanding of their disease, and even when facing the same disease, different patients may have totally different perceptions. To be specific, negative disease perception can intensify patients’ adverse emotional responses, undermine their confidence in recovery, and reduce their treatment adherence. This not only affects their self - management ability regarding the disease but also hinders the improvement of the prognosis, ultimately lowering their QoL. On the contrary, positive disease perception can enhance patients’ psychological state, correct cognitive biases and negative behaviors, strengthen self - management efficacy, and optimize the disease prognosis, thus improving their QoL (57). Therefore, in clinical working practice, assessing and intervening in patients’ IP should form an integral part of psychological oncology care. It is necessary to establish an integrated online and offline health education system. Offline sessions include regular lung cancer chemotherapy patient support groups, while online initiatives feature a WeChat public platform delivering short videos on chemotherapy procedures, side-effect management, and successful recovery cases. Empower patients with knowledge to help them build a positive cognitive framework about their illness, thereby improving their QoL.

The findings of this study indicate that the fear of disease progression (FoP) plays a significant mediating role between IP and QoL among lung cancer chemotherapy patients. This means that IP indirectly impacts patients’ QoL by influencing their levels of FoP. Specifically, when patients have a negative perception of their illness, they tend to overfocus on the disease itself and over-exaggerate its negative consequences, which can lead to heightened levels of fear of disease progression (FoP). When FoP reaches or exceeds the clinical significance threshold of 34 points, patients may experience chronic anxiety, depression, and insomnia, which can directly undermine their QoL (58). Furthermore, elevated FoP levels can compromise patients’ treatment adherence and compliance, leading to resistance against treatment plans and unwillingness to cooperate (59). This can impair clinical efficacy and prognosis, further reducing patients’ QoL (60). Conversely, a positive IP can enhance patients’ self-management capacity and help establish a sense of control, thereby reducing FoP levels. Interestingly, moderate levels of FoP may even serve as a motivator for patients to actively cope with their illness, increasing their awareness of preventive healthcare and improving treatment adherence (59, 61), which can enhance their QoL. This process exemplifies the dynamic interplay between cognition, emotion, and behavior within the self-regulation commonsense model, thereby confirming the model’s core hypothesis that IP influences health outcomes through emotional responses such as FoP. Therefore, to prevent negative IP from impacting QoL through FoP, a systematic, personalized, and multi-tiered assessment and intervention process should be developed in clinical practice. During the patient’s chemotherapy journey, especially in the initial phase and at key assessment points, FoP levels should be closely monitored. For high-risk patients with FoP scores ≥34 points, timely interventions such as individualized cognitive-behavioral therapy (CBT), mindfulness-based stress reduction, and psychoeducation should be provided. A key priority should be placed on correcting catastrophic thinking to prevent negative emotions from worsening. Additionally, establish a multidisciplinary patient support alliance. organize regular communication activities inviting recovered patients to s hare their cancer-fighting experiences and discuss symptoms, emotional changes, and coping strategies during chemotherapy. This peer support enables patients to gain psychological support and practical insights, thereby alleviating fear of the unknown (62).

## 5 Conclusion

This study is the first to validate the mediating role of FoP between disease perception and quality of life in a cohort undergoing chemotherapy for lung cancer, revealing a potential pathway whereby negative disease cognition impairs quality of life by exacerbating FoP. This discovery extends the application of the self-regulation common sense model to this population, providing empirical evidence for targeted psychological interventions to block negative emotional transmission and enhance patients’ quality of life. The findings underscore the importance of early identification and management of disease-related fear in clinical practice, providing a crucial entry point for optimizing holistic care strategies for patients undergoing chemotherapy for lung cancer.

## 6 Limitations

Our current study has several limitations. Firstly, the cross-sectional design only allows data collection at a specific point in time, precluding the determination of causal relationships or temporal sequences between variables. Prospective longitudinal studies are warranted. Secondly, owing to resource constraints, the sample was restricted to lung cancer chemotherapy patients from one local hospitals, potentially limiting the generalizability of our findings. Future work should involve multicenter studies recruiting a larger cohort of lung cancer chemotherapy patients. Additionally, factors such as patients’ psychological resilience, anxiety, social support, coping style, and depression may influence QoL. The exclusion of these variables in the present study may limit our understanding of the relationship between IP and QoL. Future research designs should incorporate these variables to more accurately elucidate the potential mechanisms linking IP, FoP, and QoL. Finally, this study’s data collection relied on patient self-reporting, which may introduce self-assessment bias and affect the objectivity of the findings. Although the scales used were adapted for Chinese contexts, their cross-cultural validity might still influence the research outcomes. Future studies could further refine measurement tools while considering the potential impact of cultural differences.

## Data Availability

The original contributions presented in the study are included in the article/Supplementary Material. Further inquiries can be directed to the corresponding authors.
